# The mediating effect of immune markers on the association between ambient air pollution and adult-onset asthma

**DOI:** 10.1038/s41598-019-45327-4

**Published:** 2019-06-19

**Authors:** Nahid Mostafavi, Ayoung Jeong, Jelle Vlaanderen, Medea Imboden, Paolo Vineis, Debbie Jarvis, Manolis Kogevinas, Nicole Probst-Hensch, Roel Vermeulen

**Affiliations:** 1 Division of Environmental Epidemiology, Institute for Risk Assessment Sciences, Utrecht University, 3584 CM Utrecht, the Netherlands; 2 Swiss Tropical and Public Health (TPH) Institute, Basel, Switzerland; 3 Department of Public Health, University of Basel, Basel, Switzerland; 4 Italian Institute for Genomic Medicine (IIGM), Turin, Italy; 5 Medical Research Council-Public Health England Centre for Environment and Health, Department of Epidemiology and Biostatistics, Imperial College London, London, United Kingdom; 6 Department of Public Health Sciences, King’s College, London, UK; 7 ISGlobal, Barcelona, Spain; 8 Julius Center for Health Sciences and Primary Care, University Medical Center Utrecht, Utrecht, The Netherlands

**Keywords:** Diagnostic markers, Chronic inflammation

## Abstract

We aim to investigate to what extent a set of immune markers mediate the association between air pollution and adult-onset asthma. We considered long-term exposure to multiple air pollution markers and a panel of 13 immune markers in peripheral blood samples collected from 140 adult cases and 199 controls using a nested-case control design. We tested associations between air pollutants and immune markers and adult-onset asthma using mixed-effects (logistic) regression models, adjusted for confounding variables. In order to evaluate a possible mediating effect of the full set of immune markers, we modelled the relationship between asthma and air pollution with a partial least square path model. We observed a strong positive association of IL-1RA [OR 1.37; 95% CI (1.09, 1.73)] with adult-onset asthma. Univariate models did not yield any association between air pollution and immune markers. However, mediation analyses indicated that 15% of the effect of air pollution on risk of adult-onset asthma was mediated through the immune system when considering all immune markers as a latent variable (path coefficient (β) = 0.09; 95% CI: (−0.02, 0.20)). This effect appeared to be stronger for allergic asthma (22%; β = 0.12; 95% CI: (−0.03, 0.27)) and overweight subjects (27%; β = 0.19; 95% CI: (−0.004, 0.38)). Our results provides supportive evidence for a mediating effect of the immune system in the association between air pollution and adult-onset asthma.

## Introduction

Asthma is a chronic inflammatory disease of the airways which has a significant impact on quality of life^1^. Current research suggests a complex etiology for asthma^2^, and there is emerging evidence that air pollution is one of the important environmental factors that may be involved in disease pathogenesis^3^. However, previous studies are inconsistent with respect to the role of air pollution in the development of asthma in adults^4,5^.

The mechanism by which air pollution may lead to asthma is an active research area^6^. Exposure to air pollutants induces oxidative stress and activates inflammatory pathways^7,8^, which have also been implicated in asthma development^9^. Research has also indicated that interleukins (IL), tumor necrosis factor (TNF), and pro-inflammatory cytokines may have an active role in the recognition of air pollutants and subsequent inflammatory response^10^.

Several studies have reported on associations between long-term exposure to air pollution and chronic changes in immune markers^8,11,12^. For example, in the ESCAPE study, living close to busy traffic was found to be associated with increased C-reactive protein concentrations^13^. In another study long-term exposure to NO_x_ was reported to be associated with decreased levels of circulating IL-8, IL-10, IL-2, and TNF-α concentrations^8^. Human airway cells or bronchial epithelial cells have been shown to release anti-inflammatory cytokines and chemokines such as IL-10 or IL-8 upon incubation of ambient air particles^14,15^. Altogether, both observational and experimental studies have provided supportive evidence for the interaction between air pollutants and the immune-system in particular inflammatory pathways.

As air pollution has been linked to inflammatory responses and as these responses have been linked to adult asthma it would follow that the observed effect of air pollution on asthma is potentially mediated by the immune system. There is however a dearth of studies that has investigated this. As immune markers resembling inflammatory responses are pleiotropic it could be argued that such analyses should not be done on single immune markers but on a combined measure of the immune response. We therefore used partial least square path modeling (PLS-PM) to identify the relationship between adult-onset asthma and air pollutants and the possible mediating effect of immune markers simultaneously. PLS-PM enables us to combine information from multiple air pollution and immune markers by using a latent structure pattern for both the air pollutants (i.e. *air pollution*) and immune-markers (i.e. *immune-modulation*). We considered long-term exposure to multiple air pollution markers (particulate matter (PM) smaller than 2.5 µm (PM_2.5_), and smaller than 10 µm (PM_10_), nitrogen dioxide (NO_2_), ultra-fine particulates (UFP) [based on two metrics: particle number concentration (PNC) and lung deposited surface area (LDSA)]) and a panel of 13 immune markers in peripheral blood samples collected from 140 adult cases (asthma diagnosis after the age of 16) and 199 controls using a nested-case control design. Previous analysis in this cohort has shown an association between air pollutants and adult-onset asthma, in particular UFP^16^.

## Results

**Figure 1:**
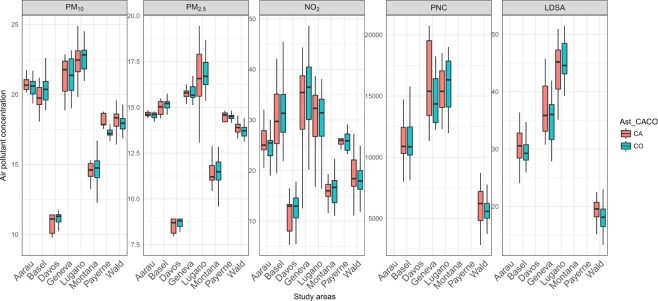
Box plots of distribution of air pollution concentrations by case (CA; red) and control (CO; green) per study area. Each panel shows one air pollution marker; PM_10_, PM_2.5_ (PM_2.5_ and PM_10_ both, estimated from the PolluMap dispersion models; µg/m^3^), NO_2_ (estimated from LUR model; µg/m^3^), PNC (particle number concentration; particles/cm^3^), and LDSA (lung deposited surface area; µm^2^/cm^3^). Horizontal lines correspond to medians, and boxes to the 25th–75th percentiles; whiskers extend to data within the interquartile range times 1.5.

Our study population consists of 339 non-smoking adults (median age 58 years) from 8 areas in Switzerland of whom 140 participants developed asthma after the age of 16 years old (Table 1). There were no differences between cases and controls for any of the baseline variables investigated except BMI and study area. Median BMI for cases (26 Kg/m^2^; [P_25_-P_75_]: [23.1, 29.6]) was higher than controls (24 Kg/m^2^; [P_25_-P_75_]: [22.4, 27.2]). In Fig. 1, we show the distribution characteristics of different air pollutants by study area. For all air pollutants, substantial variation was observed between (particularly PM_10_, PM_2.5_) as well as within (particularly NO_2_, PNC, and LDSA) areas. UFP exposures (both PNC and LDSA) were only measured and estimated in 4 of the 8 areas (Basel, Geneva, Lugano, and Wald) and were available for a subset of samples (76 cases, 113 controls). Median concentrations of air pollution were generally higher in the cities of Geneva and Lugano and were lower in the more rural and smaller communities Davos (for PM_10_, PM_2.5_ and NO_2_ concentrations) and Wald (for PNC and LDSA). We observed high correlations between air pollutants within the study population (Fig. S1) especially between PNC and LDSA (r = 0.95) and between PM_10_ and PM_2.5_ (r = 0.95).

**Table 1:** Characteristics of study participants.

Characteristic	Control (N = 199)	Cases (N = 140)	Differences P-value^a^
**Sex (N sample (%))**			
Male	96 (48)	53 (38)	
Female	103 (52)	87 (62)	0.074
BMI (Kg/m^2^; median and P_25_-P_75_)	24 (22.4, 27.2)	26 (23.1, 29.6)	0.002
Age (years; median and P_25_-P_75_)	57.1 (48.7, 64.8)	59.5 (48.6, 67.9)	0.26
**Education (N sample (%))**			
Primary school	2 (1)	4 (3)	
Secondary school, middle school, or apprenticeship	126 (63)	87 (62)	
Technical college or university	71 (36)	49 (35)	0.44
**Physical activity** ^**b**^			
Insufficiently active	40 (20)	38 (27)	
Sufficiently active	156 (80)	101 (72)	0.18
**Season (N sample)**			
1: spring (21/3-20/6)	52 (26)	33 (24)	
2: summer (21/6-20/9)	58 (29)	39 (28)	
3: autumn (21/9-20/12)	48 (24)	30 (22)	
4: winter (21/12-20/3)	41 (21)	37 (27)	0.63
**Area (N sample)**			
Basel	26 (13)	19 (14)	
Wald	56 (28)	19 (14)	
Davos	21 (11)	13 (9)	
Lugano	21 (11)	27 (19)	
Montana	21 (11)	19 (14)	
Payerne	12 (6)	5 (4)	
Aarau	27 (14)	20 (14)	
Geneva	15 (8)	18 (13)	0.02

^a^P-value for difference was calculated using the chi-squared test for categorical baseline variables and the student’s t-test for continuous variables.^b^ Sufficiently active: either moderate physical activity ≥150 min/week, vigorous physical activity ≥60 min/week, or combined duration (duration of moderate physical activity + 2 × duration of vigorous physical activity) ≥150 min/week; Insufficiently active: otherwise.

Exposure to UFP was associated with an increased risk of adult-onset asthma as previously reported (Jeong *et al*., 2018) (Table 2). The odds ratio (OR) was 3.23 [95% CI (1.62, 6.43)] for one unit increase in natural log PNC, and 5.22 [95% CI (2.16, 12.6)] for one µm^2^/cm^3^ increase in natural-logarithm of LDSA (Table 2). These associations remained similar when not correcting for the effect of BMI in the model (Table 2). The odds ratio (OR) was 3.18 [95% CI (1.65, 6.40)] for one particles/cm^3^ increase in natural-logarithm of PNC, and 5.26 [95% CI (2.28, 12.74)] for one µm^2^/cm^3^ increase in natural-logarithm of LDSA. No associations were identified for any of the other air pollution metrics.

**Table 2:** Association between air pollution and adult-onset of asthma using random-effect logistic regression analysis.

Air pollution metric^a^	Adjusted for BMI		Not adjusted for BMI	
	P-value	OR^b^ [95% CI]	P-value	OR^b^ [95% CI]
PM_10_	0.83	1.17 [0.29, 4.68]	0.36	1.62 (0.58, 4.71)
PM_2.5_	0.88	1.13 [0.24, 5.33]	0.41	1.63 (0.51, 5.35)
NO_2_	0.37	1.37 [0.69, 2.76]	0.09	1.65 (0.94, 2.94)
PNC	0.001	3.23 [1.62, 6.43]	0.001	3.18 (1.65, 6.40)
LDSA	0.0002	5.22 [2.16, 12.6]	0.0001	5.26 (2.28, 12.74)

Note: models adjusted for age, sex, education level, and study area as random effect.

^a^All air pollution metrics have been natural log-transformed (N = 338 for PM_10_, N = 339 for NO_2_ and PM_2.5_; N = 189 for PNC and LDSA).

^b^Odds ratio (OR) for adult-onset asthma per one unite increase in the natural-logarithm of each air pollutants.

**Figure 2:**
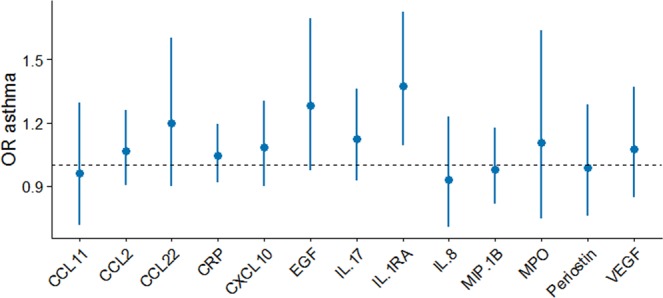
Odds ratios (OR) and 95%-confidence intervals for adult-onset asthma per IQR increase in the natural-log of each immune markers. IL-1RA was associated (p-value = 0.01, FDR = 0.08) with risk of adult-onset asthma.

Higher concentrations of IL-1RA, EGF, CCL22, CCL2, CRP, CXCL10, IL-17, and MPO were associated with an increased risk of adult-onset asthma (Fig. 2, Table S1). The strongest association was seen for IL-1RA and adult-onset asthma with a corresponding OR of 1.37 [95% CI (1.09, 1.73); FDR = 0.08] for IQR (224.84 pg/mL) increase in natural-logarithm of IL-1RA. Noteworthy were the results for EGF OR = 1.28 [95% CI (0.97, 1.96)] and CCL22 OR = 1.20 [95% CI (0.9, 1.60)] which did not reach the FDR threshold (<0.2). Interestingly, CRP OR = 1.05 [95% CI (0.92, 1.19)] showed no clear association with adult-onset asthma. (Fig. 2, Table S1).

**Figure 3:**
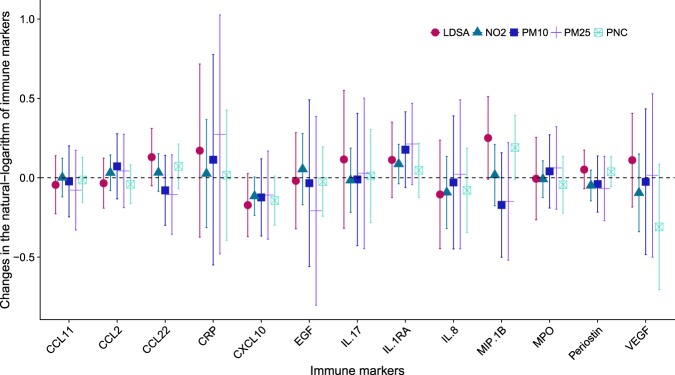
Effect estimates and 95%-confidence intervals for changes in the natural-logarithm of immune markers in per unit increase in the natural-logarithm of air pollution markers (N = 338 for PM10, N = 339 for NO_2_ and PM_2.5_; N = 189 for PNC and LDSA).

Multiple air pollutants were associated with perturbations in immune markers although none of these associations reached the threshold (FDR < 0.2) (Fig. 3). Higher concentration of IL-1RA were associated with higher exposure to all air pollutants. MIP1-β showed positive association with PNC and LDSA, and negative association with PM_2.5_ and PM_10_.

### Partial least square path modeling (PLS-PM)

**Figure 4:**
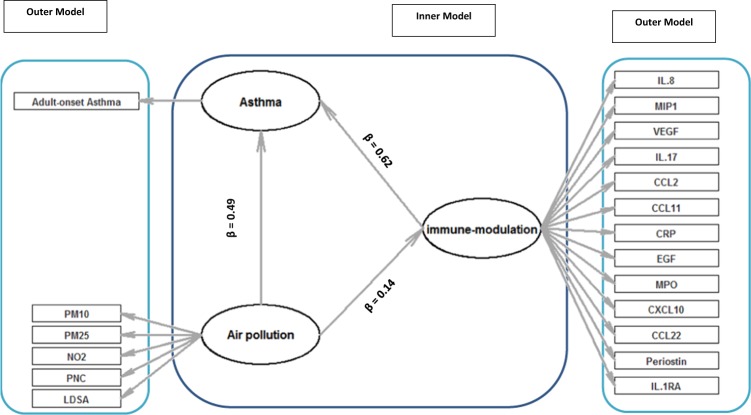
Path diagram indicating the conceptual model behind the relations among latent variables and their manifest variables. Rectangles refer to the manifest variables (outer model) and the ellipses refer to the latent variables (Inner model). Arrows show assumed causations among the variables (either latent or manifest), and the direction of the arrow defines the assumed direction of the relation. Path coefficients (β’s) indicate the quantification of the relationship between latent variables. Corresponding ORs for the one unit increase in immune-modulation and air pollution on asthma are 1.9 and 1.6, respectively.

Figure 4, shows PLS path diagrams of the latent variables *immune-modulation* and *air pollution* for adult-onset asthma. We conducted PLS-PM on the subset of data where UFP estimates were available (76 cases, 113 controls). In the measurement (outer) model, the loading and the paths revealed a quantitative relationship between the latent and observed variables. All air pollution markers had positive effects on the latent variable *air pollution* and each explained around 86–96% variance of the corresponding latent variable (Fig. 4, Table S2). In the block of *immune-modulation*, IL-1RA, CRP, VEGF, MIP1-β, and CCL22 explained the highest variability with a positive effect on the corresponding latent variable (each more than 40%; Fig. 4, Table S2). Latent variables *air pollution* and *immune-modulation* were constructed as linear combinations of corresponding observed variables using loadings in Table S2 as weights. In the structural (inner) model, the relationships between the latent variables were quantified with the standardized path coefficients (β) (Fig. 4). *Air pollution* had a positive effect on *asthma* (β = 0.49; 95% CI (0.18, 0.82) OR = 1.6; for one-unit increase in the *air pollution latent normalized structure*); Table 3) and on *immune-modulation* (β = 0.14; 95% CI (−0.27, 0.32); Table 3). The association between *Immune-modulation* and *asthma* was positive (β = 0.62; 95% CI (0.29, 0.98); OR = 1.9; for every one-unit increase in the *immune-modulation*; Table 3). The overall positive association of *air pollution* with *asthma* can be partialy explained by *immune-modulation (path coefficient* = *0.58)*. This overal effect can be calculated by summing up the indirect (path coefficients = 0.14 × 0.62) and direct (path coefficient = 0.49) effect of *air pollution* on *asthma*.

**Table 3:** Partial least square path modeling analysis for the relationships between latent variables.

	Path Coefficients (Using data)	Estimated p-value	R^2^	Path Coefficients (Using 200 data set in Bootstrap)	SE	95 LCI	95 UCI	OR^b^
Direct Effect								
β_Air pollution-> immune-modulation_	0.14	0.057	0.02	0.08	0.20	−0.27	0.32	—
β_Air pollution -> Asthma_	0.49	0.002	0.11^a^	0.49	0.16	0.18	0.82	1.6
β_immune-modulation -> Asthma_	0.62	0.0004		0.62	0.18	0.29	0.98	1.9

^a^For logistic regression we calculated pseudo-Nagelkerke R^2^.

^b^Odds ratios (OR) for adult-onset asthma per one unit increase in the corresponding latent variable.

The results of the bootstrap sampling indicated that the original coefficient path value and the one obtained from the bootstrap are close (Table 3). The average coefficient of determination R^2^ value for two latent variables in our analysis, *immune- modulation*, and *asthma* (pseudo-Nagelkerke R^2^), was found to be 0.02 and 0.11 (Table 3) respectively. This indicated that *immune-modulation* and *air pollution* together explained 11% of variation in *asthma*.

Our results indicated that of the total effect of *air pollution* on *asthma* 15% (ratio of an indirect effect on total effect) is explained by the indirect effect of *immune-modulation* although this effect did not reach the threshold (P-value < 0.05) (path coefficient = 0.09, Z-statistics = 1.64, 95% CI (−0.02, 0.20).

### Sensitivity analysis

Sensitivity analysis indicated that the associations of LDSA and PNC with adult-onset of asthma were generally robust to the set of confounders considered. The association of IL-1RA with adult-onset of asthma that we observed in the main analysis was recovered in all of the sensitivity analysis. The effect was weaker when we included BMI in the model as a confounder or in the analysis stratified by BMI status (Fig. S2B). The association between air pollution and immune markers did not change when we stratified analysis by BMI or included BMI as a confounder in the model (Fig. S3). Results of PLS-PM among the overweight subgroup indicated a stronger association between *Immune-modulation* and *asthma* (β = 0.66; 95% CI (0.18, 1.18); OR = 1.93; for one-unit increase in the *immune-modulation*; Table S5) as well as between *air pollution* and *Immune-modulation*, β = 0 0.29; 95% CI (−0.39, 0.48); Table S5), compared to the main analysis. This analysis also showed that a higher proportion (27%) of the total effect of *air pollution* on a*sthma* is explained by the indirect effect of *immune-modulation* (path coefficient = 0.19, Z-statistics = 1.92, 95% CI (−0.004, 0.38)) in comparison with the main analysis (15%; 95% CI (−0.02, 0.20)).

The magnitude of the risks (ORs) of immune markers associated with adult-onset asthma was often stronger for allergic asthma than non-allergic asthma (Fig. S2C). In the allergic group, we observed a positive association of CCL22 and IL-1RA with adult-onset asthma. The odds ratios were 1.55 [95% CI 1.07, 2.23] for IQR (245.6 pg/mL) increase in natural-logarithm of CCL22, and 1.38 [95% CI 1.06, 1.80] for IQR (220.4 pg/mL) increase in natural-logarithm of IL-1RA. The associations of EGF and Periostin with adult-onset asthma were positive but weak. The odds ratios were 1.36 [95% CI 0.97, 1.90] for IQR (56.8 pg/mL) increase in natural-logarithm of EGF, and 1.34 [95% CI 0.96, 1.88] for IQR (33862.4 pg/mL) increase in natural-logarithm of Periostin. For allergic asthma, PLS-PM also indicated a stronger association between *Immune-modulation* and *asthma* (path coefficient = 0.82 (OR = 2.27; for one-unit increase in the *immune-modulation*), P-value = 0.0003; Table S4). This analysis also showed that a higher proportion (22%) of the total effect of *air pollution* on *asthma* is explained by the indirect effect of *immune-modulation* (path coefficient = 0.12, Z-statistics = 1.6, P-value = 0.1) in comparison with the main analysis (15%).

## Discussion

We investigated the relationship between exposure to air pollution, immune markers, and adult-onset asthma using data from the Swiss Cohort Study on Air Pollution and Lung and Heart Diseases in Adults (SAPALDIA). Previous studies have investigated the associations of immune markers with either air pollution or adult-onset asthma separately, which limits causal interpretation. The main aim of the current study was to better understand the biological mechanism linking air pollution exposure to adult-onset asthma and to study if immune-markers mediate this association.

We previously published on the positive association between biannual mean exposure to UFP (both PNC and LDSA) with adult-onset asthma^16^. In these extended analyses, we observed non-significant positive associations between PM_10_, PM_2.5_ and NO_2_ and adult-onset asthma. Additionally, we observed an increased risk of asthma with elevated concentrations of IL-1RA, and to some extend with EGF, and CCL22. Of these IL-1RA achieved Benjamini-Hochberg (BH-FDR) < 0.2 in main analysis as well as in sensitivity analyses. We observed limited evidence for a univariate association between air pollution and immune markers. IL-1RA was positively associated with all air pollutants and higher concentration of MIP1-β and CRP and lower concentration of IL-8 was observed with UFP exposures. Our study further suggested that 15% of the total effect of air pollution on adult-onset asthma is explained by the indirect effect of immune markers. These associations were robust after inclusion of different confounders.

A previous review of epidemiological studies reported that the evidence to support a causal relationship between air pollution and adult-onset asthma was inconsistent and identified that there was a need for large-scale cohort studies and the inclusion of local-scale traffic pollutants like UFP^17^. Although our study population is still modest in size (N = 339, Cases = 140), we were able to study its association with two metrics of UFP (LDSA and PNC). The results indicated that UFP contributes to increases in adult-onset asthma independently of other potential risk factors. The association, which we reported previously, with UFP in our study was in contrast with a recent study that assessed long-term exposure to UFP in Toronto, where a city-specific LUR model was developed and linked to health registry data of 1.1 million adult city residents. Within this study, association between UFP exposure and respiratory disease incidence, including adult-onset asthma was not reported^18^. A study of young adult Italians (aged 20–44 years) reported a positive (albeit weak) relationship between NO_2_ exposure and asthma prevalence (OR 1.13, 95% CI 0.98–1.32 per 18.3 µg/m^3^)^19^. Further, a study of 12 177 Australian females reported no association between the 3-year mean annual NO_2_ and asthma prevalence (RR: 0.97, 95% CI: 0.86–1.10)^20^.

There is growing evidence linking systemic inflammation in the pathogenesis of asthma^9^. Additionally, the higher susceptibility of individuals with asthma to air pollution may be attributable to a combination of local inflammation, and oxidative stress^21^. The current study investigated a relatively wide panel of inflammatory biomarkers to evaluate the effects of long-term exposure on systemic inflammatory status in adults. Our results are consistent, in terms of direction, with other studies on immune markers measured in adults showing elevated levels of CRP, and MIP1-β^12,13,22^ and decreased levels of IL-8 and EGF^8,22^ in relation to long-term exposure to air pollution. These markers were not noteworthy in our study. Our study also provides evidence for perturbation of plasma concentration of IL-1RA in association with exposure to air pollution in particular UFP. IL-1RA is a pleiotropic cytokine which has an anti-inflammatory effect. By suppressing the production of multiple pro-inflammatory cytokines (including TNF-α, IL-1β, IL-6, and IL-8) it regulates the progression of the immune response^23,24^. To date, this immune marker has not been reported in previous studies on immune markers and air pollution.

Our study shows that plasma concentrations of IL-1RA, EGF, and CCL22 were elevated in asthma patients compared to the control group. This is in line with previously reported increases in expression of IL-1RA in asthmatic airway epithelium^25^. Moreover, IL-1, of which IL-1RA is the receptor antagonist, is a critical mediator of the inflammatory process of multiple diseases, including asthma^26^. Therefore, it is reasonable to postulate that some of these markers may be responsible, at least in part, for a dysregulated inflammatory response in asthma^26^.

In addition to univariate regression analysis, we applied PLS path modeling that is designed to test for associations in a network of causal relationships. This approach overcomes statistical limitations by combining information from multiple variables rather than assessing them one by one. We focused on PLS path modeling in our analysis because we were interested in identifying to what extent the association between air pollution and asthma can be explained by air pollution induced immune modulation. Moreover, PLS path modeling enabled a joint assessment of the full set of immune biomarkers and air pollution exposures and does not suffer from instability in estimates and failure to converge due to multicollinearity. Our study also demonstrated that PLS path modeling is effective in assessing the total effects and relative importance of various pathways, as reflected in the latent variables, on adult-onset asthma. As univariate analysis indicated a stronger association of UFP with asthma, we also tested the mediating effect of immune markers when only using PNC and LDSA in the outer model of PLS-PM (Table S3). Interestingly, this model indicated a similar mediating effect of UFP (as measured by PNC and LSDA) on adult-onset asthma (15%). Sensitivity analyses using a two-stage regression approach to correct first for confounding factors and then apply a PLS-PM on the residuals lead to essential similar results (not shown).

Epidemiological studies have shown that obesity represents a pro-inflammatory state^27^ which itself may increase susceptibility to air pollution by increasing the inflammatory response. Being overweight has been found to increase susceptibility to the respiratory effects of air pollution^28–30^. In our analysis, the effect of immune markers on asthma weakened when we corrected for the effect of BMI. As BMI may be on the causal path, inclusion of BMI in the model will in that scenario lead to an underestimation of the effect of air pollution on immune markers. We therefore put more emphasis on the non BMI corrected analyses. Interestingly, PLS mediation analysis among the overweight sub group showed a stronger mediation effect of the immune system on the relation between air pollution and asthma (27% versus 15% in the overall analyses). This result may provide additional support to the hypothesis that obesity may indeed increase susceptibility to air pollution.

The strengths of the current study include a relatively large and well-characterized study population, individual assessment of exposure to long-term air pollution based on locally optimized models, precise measurements of plasma immune markers concentrations, and evaluation of combined mediating effect of pleiotropic immune biomarkers using PLS-PM.

Our study was also subject to some (potential) limitations. Prerequisites for mediation analysis are an association between exposure (e.g. air pollution) and outcome (e.g. asthma), exposure and mediator (e.g. an immune marker), and an association between mediator and asthma. In our analyses on an individual marker level these prerequisites were not met: there was a mis-alignment in the immune markers that were associated to air pollution and those that were associated to asthma. However, when we view the total panel of immune markers that was included in our study as a proxy for the immune system as a whole, prerequisites for mediation analysis on group level were met. Furthermore, we assumed that the weak associations that we observed between air pollution, asthma and individual immune markers was at least partly explained by measurement error of these markers. By focusing on the underlying latent structures of the immune markers, the impact of this measurement error is reduced, which likely contributed to our ability to observe an association between air pollution and asthma and the latent structure of the immune markers.

Even though we investigated a relatively large set of immune markers in relation to air pollution exposure and adult-onset asthma, these 13 immune markers may not fully represent all aspects of the immune system. As such the effect of the immune system on adult-onset asthma may be underestimated as well as its mediating effect of air pollution on asthma. Future studies should attempt to explore a broader range of inflammatory cytokines in order to expand our understanding of underlying mechanisms and the interactions between cytokines/chemokines in the air pollution/asthma inflammatory response.

This study is also limited by examining not incident but prevalent asthma cases. Asthma is a complex chronic disease that can be unnoticed for long time, grow out, and resurface, complicating accurate definition of the disease onset. Therefore this study is cross-sectional in nature, making it difficult to draw causal inference, although we restricted to the adult-onset asthma that is less susceptible to reverse causation. Additionally, the case definition of asthma in our study was based on self-report that may be prone to recall bias.

Air pollution estimates used in our analyses reflect exposure to UFP at the time of blood sample collection and therefore do not reflect a precise estimation of participants’ exposure before they had developed asthma. However, work in The Netherlands has suggested that current levels of UFP are relevant for past exposures at least in relative sense^31^. Occupational exposure may contribute to asthma development in adults and it is conceivable that occupational asthma may constitute a distinct asthma phenotype in adults^32^. In our study, however, the odds of adult-onset asthma did not differ by occupational exposure to vapors, gas, dust, and fumes (Fisher’s test p-value > 0.99).

## Conclusion

The current study adds to the growing body of evidence demonstrating the inflammatory effects of exposure to long-term air pollution. Furthermore, the current study provides supportive evidence for the mediatory effect of the immune system in the association between air pollutants (in particular UFP) and adult-onset asthma.

## Material and Methods

### Study population

SAPALDIA is a population-based study that was initiated in 1991 to investigate the effect of long-term exposure to air pollution on the respiratory health of the adult Swiss population (18–60 years, N = 9651). As previously described in detail^33,34^, the study comprised of eight distinct rural and urban areas covering the environmental and geographic diversity of Switzerland (Basel, Geneva, Davos, Aarau, Payerne, Montana, Wald, and Lugano). A subset of baseline participants completed two follow-up assessments in 2002–2003 (SAPALDIA2, n = 8047) and in 2010–2011 (SAPALDIA3, n = 6088). In both follow-up assessments blood was drawn and stored at −80 °C in the SAPALDIA biobank. The present study included individuals selected among participants of SAPALDIA3. Information on age, sex and level of education were provided by self-reported questionnaire data collected at the study examinations. Smokers were excluded so that all participants had not smoked for at least one year before blood was drawn at SAPALDIA2.

This study complies with the Declaration of Helsinki principles and all participants provided informed consent. The overall SAPALDIA study protocol was approved by the Swiss Academy of Medical Sciences and the regional committees for each study center.

### Asthma definition

Asthma cases (N = 203) were defined as participants who reported asthma in SAPALDIA3. In this study we limited our analyses to adult-onset asthma, which refers to participants who reported the onset of asthma at age 16 years or older (140 out of 203 cases). Controls (N = 199) were randomly sampled among the participants who never reported asthma or asthma related symptoms since the first follow up in SAPALDIA1. Specifically, controls were selected among those who never reported any of the following: self-reported asthma; physician-diagnosed asthma; asthma attack in the last 12 months; current asthma medication; wheezing without cold; or three or more asthma-related symptoms in the last 12 months (breathless while wheezing; attack of shortness of breath at rest or after exercise; or woken up with a feeling of chest tightness or shortness of breath).

Participants were tested for atopy at baseline via skin-prick test. Atopy was defined as developing a wheal of diameter 3 mm or larger to one or more of eight common inhalant allergens (cat, timothy grass, parietaria, birch, house-dust mite, Alternaria tenuis, Cladosporium herbarum, and dog).

### Exposure assessment

Mean biennial exposure to UFP was estimated using multi-area land use regression (LUR) models which were derived from SAPALDIA-specific measurement campaigns conducted in 2011–2012^35^. Particle number concentration (PNC) and lung deposited surface area (LDSA) were used as metrics of UFP exposure and covered 4 out of 8 SAPALDIA study areas (covering 76 cases and 113 controls)^35^. LDSA is defined as the particle surface area concentration per unit volume of air, weighted by the deposition probability in the lung and was estimated according to the International Commission on Radiological Protection (ICRP)^36^. The surface area of particles is hypothesized to be a more biologically relevant metric than particle mass or number. NO_2_ exposure was estimated by applying a recently developed European LUR model (spatial resolution 100 * 100 m) which had been derived from measurements collected in 2010^37^. We also obtained annual mean estimates of PM_2.5_ and PM_10_ in 2010 from the PolluMap dispersion models developed by the Swiss Federal Office for the Environment (spatial resolution 200 * 200 m)^38^.

### Assessment of immune markers

Serum levels of a panel of 23 immune markers were measured in blood samples collected from all subjects^39^. A Luminex screening assay was performed following the manufacturer-defined protocol. The panel included interleukin (IL) 1β, IL-4, IL-5, IL-6, IL-8, IL-10, IL-13, IL-17, IL-25, tumor necrosis factor alpha (TNF-α), C-C motif chemokine 11 (CCL11), IL1 receptor antagonist (IL1ra), CXC chemokine ligand 10 (CXCL10), epidermal growth factor (EGF), fibroblast growth factor beta (FGF-β), granulocyte colony-stimulating factor (G-CSF), melanoma growth stimulatory activity/growth-related oncogene (GRO), chemokine (C-C motif) ligand 2 (CCL2), C-C motif chemokine 22 (CCL22), macrophage inflammatory protein-1 beta (MIP-1β), vascular endothelial growth factor (VEGF), Myeloperoxidase (MPO), and periostin. Additionally, C-reactive protein (CRP) was assessed using R&D System Solid Phase Swedish ELISA. Within each analytical batch, quality control samples were run in duplicate alongside the study samples.

For 10 immune markers (interleukin IL-1β, IL-4, IL-5, IL-6, IL-10, IL-13, IL-25, TNF-α, GRO, and FGF-β) the serum concentrations were below the limit of quantification (LOQ) in more than 70% of the samples. Therefore, they were excluded from analyses. Values below the LOQ for the remaining markers were imputed using a maximum likelihood estimation procedure^40^. To allow for variations in LOQ between plates, we imputed based on plate-specific LOQ and included the plate as a predictor variable in the imputation model. The maximum percentage of imputed samples was 35% (G-CSF) and 27% (eotaxin), while other markers had less than 4% imputed values. Immune markers were natural log-transformed to approximate a normal distribution and stabilize variance.

### Statistical analysis

#### Univariate analysis

The heterogeneity of demographic and lifestyle variables between asthma cases and controls were compared using chi-square test. We performed random-effects logistic regressions for all air pollution and immune markers to assess the association with adult-onset asthma. Linear mixed-effects regression analysis was carried out to evaluate the relationship between immune markers and long-term exposure to air pollutants.

All analyses were adjusted for sex, age, body mass index (BMI) (kg/m^2^) [only for air pollution and asthma analysis], education (primary, high school, university) as fixed-effects to control for potential confounding effects. To capture heterogeneity between the study areas, city (Basel, Wald, Davos, Lugano, Montana, Payerne, Aarau, and Geneva) was included as a random-intercept in all analysis.

To capture the nuisance variation generated in the immune marker assessment, we included a random intercept for microtiter plate. Additionally, we adjusted for bench time, fasting time, Fourier-transformed venipuncture time point assuming one or two periods per day, and their multiplicative interaction terms with fasting time as potential confounders in immune marker analysis. As BMI could be on the causal path from air pollution to immune-modulation and asthma we did not adjust for BMI in the main analyses of immune markers. The set of confounders that were considered for each analysis in the current study is consistent with our previous publication^16^ using the same case-control dataset.

Statistical analyses were conducted using R version 3.4.0 (packages: *glmer*^41^ and *lme4*^41^). The resulting p-values underwent multiple testing adjustments using the false discovery rate method of Benjamini-Hochberg (BH-FDR)^42^ at the level of 0.2. A Directed acyclic graph (DAG) visualizing the causal assumptions made in our analytical plan is provided in the Supplementary Information (Fig. S4).

#### Sensitivity analysis

To assess how sensitive our findings were to variations in the confounder model, we conducted a set of additional analysis. We ran a minimally adjusted model (only age and sex included as covariates), a model in which study area was excluded, and a model in which we added season, and physical activity (insufficiently active and active) as covariates. For immune marker analysis, we ran a model in which BMI was included as a covariate and a model in which we excluded Fourier-transformed venipuncture time point from the list of covariates. In analysis of air pollution and asthma we ran a model in which BMI was not adjusted.

Additionally, we examined potential effect modification of the associations by BMI (dichotomized into Overweight [BMI ≥ 25 kg/m^2^; N = 164], or Normal weight [18 ≥ BMI < 25; N = 170]; skin prick test defined as allergic (positive test [78 cases]) or Non-allergic (negative test [62 cases]) in a further sensitivity analysis, as differences in susceptibility to air pollution by BMI and difference in reaction to air pollution by atopic status has been suggested^29,30^. Few individuals (N = 5) in our study were underweight (BMI < 18 kg/m^2^) and were therefore excluded from the stratified analysis by BMI.

#### Mediating effect of immune markers in the association between air pollution and asthma

We analyzed the potential mediating effect of immune markers on the association between air pollution and adult-onset asthma by utilizing partial least-squares path modeling (PLS-PM) within the R package *plspm*^43^. This technique relies on taking into account all relationships among unobserved or latent variables (LV), which have each been measured by several observed indicators, generally defined as observed or manifest variables (MV). PLS-PM models are formally defined by two sets of linear equations: structural (Inner) model and measurement (Outer) model. The structural model indicates the relationships among the latent variables “*immune-modulation*”, “*air pollution*”, and “*asthma*”, which are inferred from the observed immune markers, air pollutant markers, and adult-onset asthma in this study, respectively. The measurement (outer) model indicates the relationship between the latent variables and their corresponding manifest variables. In our study, the manifest variables for the latent variable of air *pollution* are based on the six air pollution markers and observed values for the latent variable of *immune-modulation* are based on 13 immune markers. The algorithm includes two steps: first is an iterative estimation of the latent construct scores; second is the final estimation of the PLS-PM coefficients. To estimate the coefficients of the PLS-PM in the final step, we analyzed the association between *asthma* and latent variables of *air pollution* and *immune-modulation* using logistic regression. To evaluate the good-ness of fit, we calculated the average coefficient of determination R^2^ value for two latent variables in our analysis, *immune- modulation*, and *asthma* (pseudo-Nagelkerke R^2^). The detailed methodology and algorithm can be found in previous publications^44,45^. In order to determine the confidence intervals for the path coefficient of *air pollution* on *immune-modulation*, a bootstrap analysis was carried out using 200 sample data sets^45^. Additionally, we tested the statistical significance of the indirect effect of air pollution on asthma^46^ to establish the mediating effect of *immune-modulation*. PLS-PM does not allow for confounders to be considered in the modeling. To address this issue, we additionally employed a two-stage regression approach. First each exposure block (immune markers and air pollution) were separately regressed on potential confounders, and second, PLS-PM was fit on the obtained residuals.

We also performed sensitivity analyses. We tested the possible mediating effect of immune markers when only using UFP (both PNC and LDSA) to reflect the LV of air pollution. We also tested the mediation effect of immune markers in the subset of allergic cases and overweight individuals.

## Supplementary information

Supplementary Information

## Data Availability

Data can be provided via sending an email to the corresponding author.
