# Supplementary material for: The mediating effect of immune markers on the association between ambient air pollution and adult-onset asthma
Source: Sci Rep. 2019 Jun 19;9:8818. doi: 10.1038/s41598-019-45327-4 (PMC6584571; doi:10.1038/s41598-019-45327-4)
Supplement: Supplementary file 1 — Supplementary Information [file 41598_2019_45327_MOESM1_ESM.pdf]

Supplementary information

The mediating effect of immune markers on the association between ambient air pollution and adult-onset asthma

Nahid Mostafavi[a†], Ayoung Jeong[b,c†], Jelle Vlaanderen[a], Medea Imboden[b,c] , Paolo Vineis[e,f], Debbie Jarvis[g], Manolis Kogevinas[h], Nicole Probst-Hensch[b,c‡], Roel Vermeulen[a,f,i‡*]

[a] Division of Environmental Epidemiology, Institute for Risk Assessment Sciences, Utrecht University, 3584 CM Utrecht, the Netherlands;

[b] Swiss Tropical and Public Health (TPH) Institute, Basel, Switzerland;

[c] Department of Public Health, University of Basel, Switzerland;

[e] Italian Institute for Genomic Medicine (IIGM), Turin, Italy;

[f] Medical Research Council-Public Health England Centre for Environment and Health, Department of Epidemiology and Biostatistics, Imperial College London, London, United Kingdom;

[g] Department of Public Health Sciences, King's College, London, UK

[h] ISGlobal, Barcelona, Spain

[I] Julius Center for Health Sciences and Primary Care, University Medical Center Utrecht, Utrecht, The Netherlands

[†] equal first authorship

[‡]equal senior authorship

[*]Corresponding author: Roel Vermeulen, Division Environmental Epidemiology, Institute for Risk Assessment Sciences, Yalelaan 2, Room 353, 3584 CM, Utrecht, The Netherlands. E-mail: r.c.h.vermeulen@uu.nl

Table S1. Association between immune markers and adult-onset of asthma (univariate analysis)

**Immune markers**	**OR [a] [95% CI]**	**P_ value**	**FDR**	**OR [b] [95% CI]**
**IL.8**	0.98 [0.9, 1.06]	0.61	0.81	0.93 [0.71, 1.23]
**MIP.1β**	1.00 [1.00, 1.00]	0.82	0.89	0.98 [0.82. 1.17]
**VEGF**	1.00 [1.00, 1.01]	0.55	0.81	1.08 [0.85, 1.37]
**IL.17**	1.03 [0.98, 1.09]	0.23	0.76	1.12 [0.93, 1.36]
**CCL2**	1.00 [1.00, 1.00]	0.44	0.81	1.07 [0.90, 1.26]
**CCL11**	1.00 [0.99, 1.01]	0.80	0.89	0.96 [0.72, 1.29]
**CRP**	1.00 [1.00, 1.00]	0.49	0.81	1.05 [0.92, 1.19]
**EGF**	1.00 [1.00, 1.01]	0.08	0.51	1.28 [0.97, 1.69]
**MPO**	1.00 [1.00, 1.00]	0.62	0.81	1.10 [0.75, 1.64]
**CXCL10**	1.01 [0.99, 1.03]	0.41	0.81	1.08 [0.90, 1.31]
**CCL22**	1.00 [1.00, 1.00]	0.22	0.76	1.20 [0.90, 1.60]
**Periostin**	1.00 [1.00, 1.00]	0.93	0.93	0.99 [0.76, 1.29]
**IL-1RA**	**1.001 [1.0004, 1.002]**	**0.01**	0.08	**1.37 [1.09, 1.73]**

[a] Odds ratio for one pg/mL increase in natural-logarithm of each immune marker

[b] Odds ratio for IQR (inter quartile range) increase in natural-logarithm of each immune marker

Models adjusted for sex, age, education, bench time, fasting time, Fourier-transformed venipuncture time point assuming one or two periods per day, and their multiplicative interaction terms with fasting time as fixed effect and study area and plate as a random-effects.

Table S2. Partial least-squared path modeling (PLS-PM) for air pollutants, immune markers and asthma. Loadings of manifest variables on latent variables.

Manifest variable	loadings
	**Air pollution**
**PM_{10}**	0.96
**PM_{25}**	0.93
**NO_2**	0.86
**PNC**	0.96
**LDSA**	0.97
	**Immune modulation**
**IL.8**	-0.005
**MIP.1β**	0.47
**VEGF**	0.52
**IL.17**	0.05
**CCL2**	-0.05
**CCL11**	0.11
**CRP**	0.65
**EGF**	0.15
**MPO**	0.33
**CXCL10**	0.24
**CCL22**	0.43
**Periostin**	0.12
**IL-1RA**	0.77
	**Asthma**
**Adult-onset asthma**	1.00

Table S3. Partial least square path modeling for the relationships between latent variables (when only using PNC and LDSA as manifest variables of air pollution)

	Path Coefficients (Using data)	Estimated p-value	R^2	Path Coefficients (Using 200 data set in Bootstrap)	SE	95 LCI	95 UCI	OR[b]
				**Direct Effect**				
$\beta_{\text{Air pollution-> immune-modulation}}$	0.15	0.04	0.02	0.08	0.20	-0.26	0.33	-
$\beta_{\text{Air pollution -> Asthma}}$	0.54	0.001	0.11[*]	0.53	0.16	0..22	0.86	1.7
$\beta_{\text{immune-modulation -> Asthma}}$	0.62	0.0004		0.62	0.18	0.28	0.98	1.9

[*] For logistic regression we calculated pseudo-Nagelkerke R^2

[b] Odds ratios (OR) for adult-onset asthma per one unit increase in the corresponding latent variable

Total effect of air pollution on asthma is 0.54+ 0.15*0.62 = 0.63

In direct effect of air pollution on asthma = 0.15*0.62 = 0.09 (Z score= 1.76; P-value= 0.07)

Ratio of indirect effect/total (VAF) is 15%

Table S4. Partial least square path modeling analysis for the relationships between latent variables (for Allergic asthma (N= 154))

	Path Coefficients (Using data)	Estimated p-value	R^2	Path Coefficients (Using 200 data set in Bootstrap)	SE	95 LCI	95 UCI	OR[b]
				**Direct Effect**				
$\beta_{\text{Air pollution-> immune-modulation}}$	0.15	0.07	0.02	0.08	0.22	-0.30	0.37	-
$\beta_{\text{Air pollution -> Asthma}}$	0.42	0.032	0.13[a]	0.42	0.20	0.04	0.82	1.53
$\beta_{\text{immune-modulation -> Asthma}}$	0.82	0.0003		0.82	0.23	0.40	1.29	2.27

[a] For logistic regression we calculated pseudo-Nagelkerke R^2

[b] Odds ratios (OR) for adult-onset asthma per one unit increase in the corresponding latent variable

Total effect of air pollution on asthma is 0.42+ 0.15*0.82 = 0.54

In direct effect of air pollution on asthma = 0.15*0.82 = 0.12 (Z score= 1.6; P-value= 0.1)

Ratio of indirect effect/total is 0.22

Goodness of fit 0.18

Table S5. Partial least square path modeling analysis for the relationships between latent variables among overweight (N=102)

	Path Coefficients (Using data)	Estimated p-value	R^2	Path Coefficients (Using 200 data set in Bootstrap)	SE	95 LCI	95 UCI	OR[b]
				**Direct Effect**				
$\beta_{\text{Air pollution-> immune-modulation}}$	0.29	0.003	0.1	0.17	0.31	-0.39	0.48	-
$\beta_{\text{Air pollution -> Asthma}}$	0.52	0.02	0.12[a]	0.52	0.23	0.09	0.98	1.7
$\beta_{\text{immune-modulation -> Asthma}}$	0.66	0.01		0.66	0.25	0.18	1.18	1.93

[a] For logistic regression we calculated pseudo-Nagelkerke R^2

[b] Odds ratios (OR) for adult-onset asthma per one unit increase in the corresponding latent variable

Total effect of air pollution on asthma is 0.52+ 0.29*0.66 = 0.73

In direct effect of air pollution on asthma = 0.29*0.66 = 0.19 (Z score= 1.92; P-value= 0.05)

Ratio of indirect effect/total is 0.27

Goodness of fit 0.2

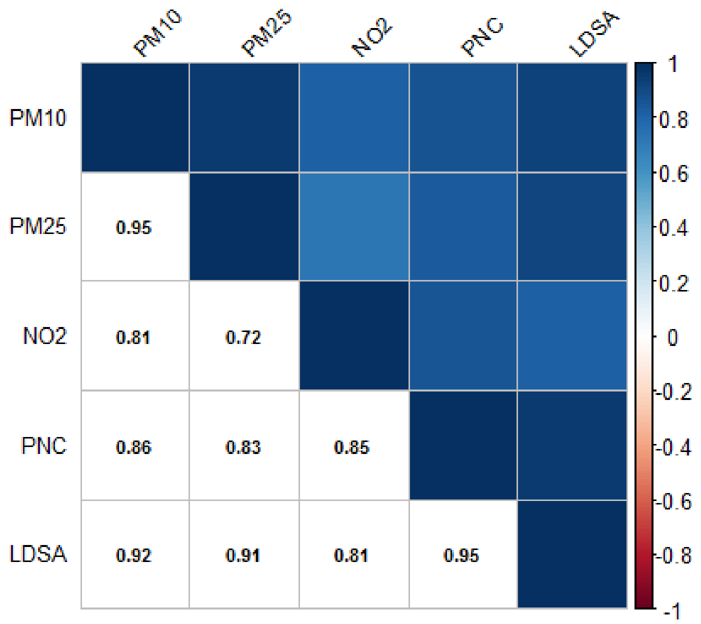

Figure S1. Pearson correlation coefficients for air pollution exposures. The color intensity of boxes indicates the magnitude of the correlation. Blue (red) color indicates the positive (negative) correlation. Numbers show the magnitude of correlation. Abbreviations are as follows: PM_{10}, $PM_{2.5}$ (PM_{10}, $PM_{2.5}$ both estimated from the PollouMap dispersion models; µg/m^3), NO_2 (NO_2, estimated from LUR model; µg/m^3), PNC (particle number concentration; particles/cm^3), and LDSA (lung deposited surface area; µm^2/cm^3).

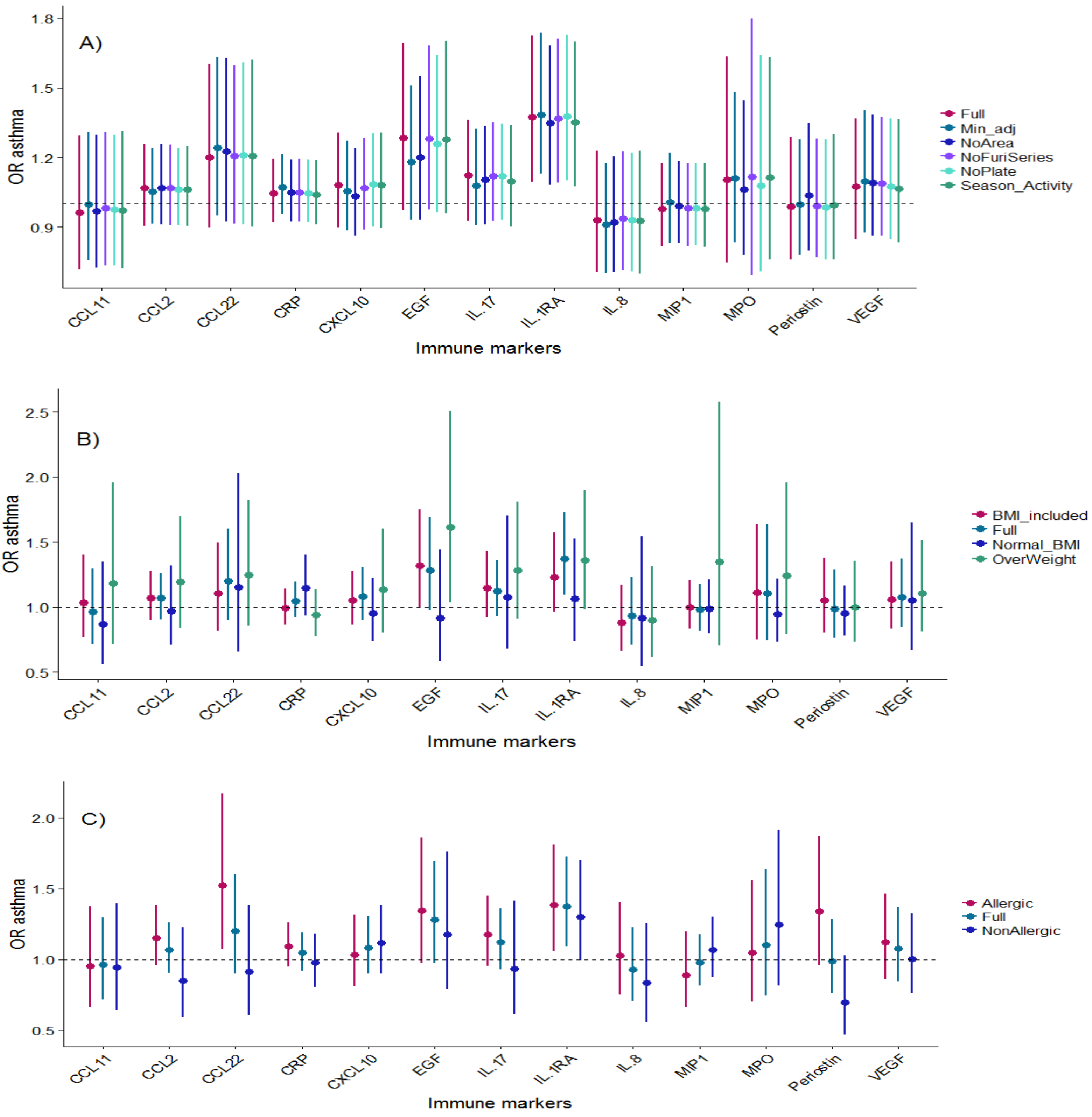
A)
B)
C)
OR asthma
Immune markers
CCL11
CCL2
CCL22
CRP
CXCL10
EGF
IL.17
IL.1RA
IL.8
MIP1
MPO
Periostin
VEGF
Full
Min_adj
NoArea
NoFuriSeries
NoPlate
Season_Activity
BMI_included
Normal_BMI
OverWeight
Allergic
NonAllergic

Figure S2. (A) Association between immune markers and adult-onset asthma for the different set of confounder adjustment including Full model adjusted for fixed-effects of sex, age, bench time, fasting time, Fourier-transformed venipuncture time point assuming one or two periods per day, and their multiplicative interaction terms with fasting time and random-effects of study area and plate. Min_adj model adjusted for age and sex. NoArea model adjusted for all variables of Full model except for study area. NoFourier-Series model adjusted for all variables of Full model except for Fourier-transformed venipuncture time point assuming one or two periods per day, and their multiplicative interaction terms with fasting time. NoPlate model adjusted for all variables of Full model except for microtiter plate. Season_Activity model adjusted for all variables of Full model and additionally for physical activity and season. (B) Association between immune markers and adult-onset asthma in the pooled population, full model additionally adjusted for BMI and stratified by BMI. (C) Association between immune markers and adult-onset asthma in the pooled population, and stratified by Allergic and none allergic.

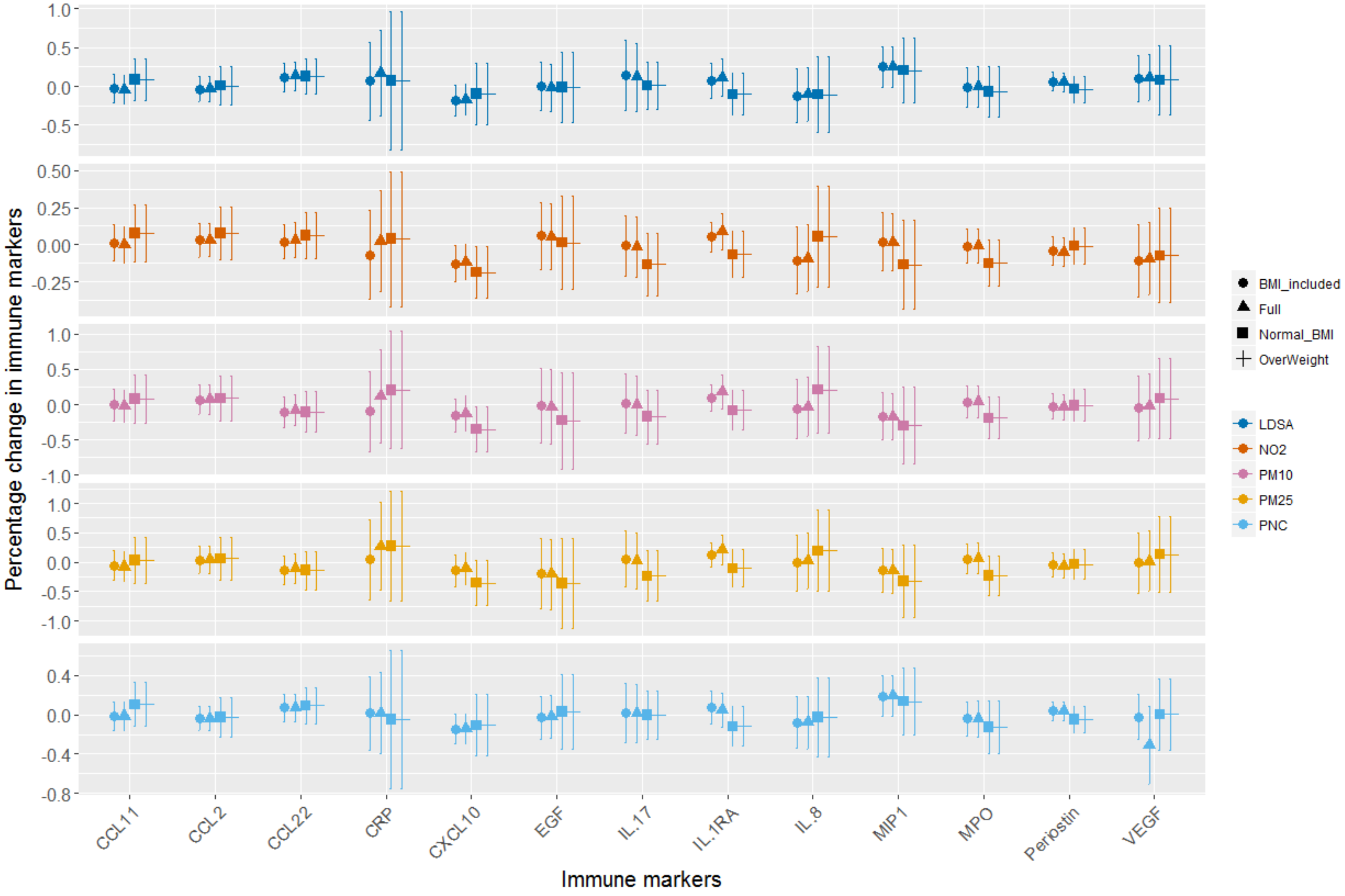

Figure S3. Association between immune markers and different air pollution exposures in the pooled population, full model additionally adjusted for BMI and stratified by BMI. Colors show the different air pollution exposure and Shapes show different model.

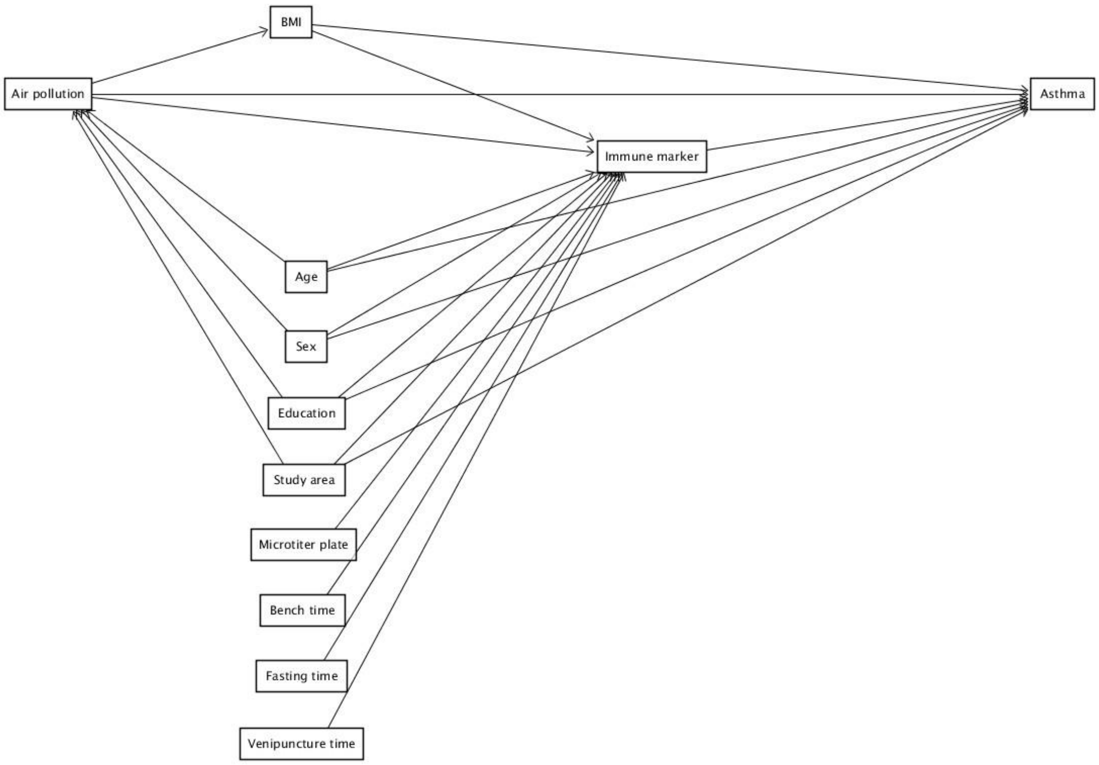

Figure S4. Directed Acyclic Graph visualising the causal assumptions of our analytical framework
